# Do Socioeconomic Inequalities Exist Within Ophthalmology and Orthoptics in the UK?: A Scoping Review

**DOI:** 10.22599/bioj.338

**Published:** 2024-01-18

**Authors:** Laura England, Anna O’Connor

**Affiliations:** 1 Manchester Royal Eye Hospital, England; 2 University of Liverpool, England

**Keywords:** Socioeconomic, Inequality, Orthoptics, Ophthalmology, United Kingdom

## Abstract

**Introduction:**

It is well documented that socioeconomic disadvantage adversely affects general health and ocular health worldwide. Within orthoptics, while clinicians recognise a relationship between socioeconomic situation and treatment outcome, no previous literature review was found to address this issue. Neither was a UK-specific literature review found to address the same issue for ophthalmology as a whole.

**Aim:**

This literature review evaluates evidence for an association between socioeconomic situation and ophthalmic/orthoptic conditions and their treatment outcomes, specifically within the UK.

**Methods:**

Keyword searches were conducted on Google Scholar and the University of Liverpool library catalogue. Results for the main analyses were limited to full papers, specific to the UK, written in English. Literature was only included from pre-2000 if more recent evidence was insufficient.

**Results:**

There is evidence of socioeconomic disadvantage being associated with the following: reduced visual acuity; reduced attendance at diabetic retinopathy screening appointments; and delayed presentation of glaucoma, cataracts, and diabetic retinopathy. However, evidence linking socioeconomic disadvantage to AMD is mixed. There is limited evidence of the increased prevalence of amblyopia and subsequent barriers to its treatment for socioeconomically underserved children. There is also evidence of a reduction in quality of life for socioeconomically underserved adults with strabismus.

**Conclusions:**

Health inequalities within ophthalmology and orthoptics are reported, but with confounding results for some conditions. Further research should explore the reasons behind the inequalities that are found and identify methods of reducing them.

## Introduction

Socioeconomic situation describes the relative advantage or disadvantage that an individual or group experiences in accessing and controlling economic, material, and social resources or opportunities (adapted from Lamnisos, Lambrianidou & Middleton 2019). Each country in the UK has its own scale to measure socioeconomic disadvantage, or ‘deprivation’, as detailed in Table 1.

**Table 1:** Socioeconomic indices used in the UK.


INDEX	DOMAINS (NUMBER OF * GROUPS SIMILAR CONCEPTS)																

	INCOME	EMPLOYMENT/UNEMPLOYMENT	HEALTH*	HEALTH & DISABILITY*	EDUCATION**	EDUCATION, SKILLS & TRAINING**	ACCESS TO SERVICES	BARRIERS TO HOUSING & SERVICES	HOUSING***	HOUSEHOLD OVERCROWDING***	PHYSICAL ENVIRONMENT***	LIVING ENVIRONMENT***	CRIME****	CRIME & DISORDER****	COMMUNITY SAFETY****	NON-CAR OWNERSHIP	NON-HOME OWNERSHIP

Scottish Index of Multiple Deprivation (SIMD)^1^	✓	✓	✓		✓		✓		✓				✓				

English Index of Multiple Deprivation (IMD)^2^	✓	✓	✓		✓			✓				✓	✓				

Welsh Index of Multiple Deprivation (WIMD)^3^	✓	✓	✓		✓		✓		✓		✓				✓		

Northern Ireland Multiple Deprivation Measures^4^	✓	✓		✓		✓	✓					✓		✓			

Townsend Deprivation Index^5^		✓								✓						✓	✓


1. SG 2020, 2. MHCLG 2019, 3. WG 2023, 4. NISRA 2017, 5. Martin 2007.

For each scale in Table 1, the domains that contribute to the overall score are almost identical, but each country uses slightly different weightings depending upon the literature specific to that country (SVP 2017)and potentially different ideas of what is important. The Townsend Deprivation Index is also included, which UK health authorities have used to assign resources due to its high level of correlation with measures of ill health (Dymond-Green 2020).

These indices can be used as a measure of socioeconomic situation to investigate its relationship with other factors. For example, it is well documented that socioeconomic disadvantage adversely affects health in the UK (Marmot 2010; PHE 2019), and the gap in healthy life expectancy (years lived in good health) between the most and least disadvantaged areas in 2018 to 2020 ranged from 12 to over 24 years (IAD 2021; NRS 2022; ONS 2022a; ONS 2022b). More disadvantaged areas in England have higher mortality rates from heart disease, lung cancer, and chronic lower respiratory diseases (PHE 2018), and in 2020, COVID-19 was the cause of death that contributed most to the gap in life expectancy between the most and least disadvantaged areas (PHE 2021a).

Health inequalities can also present during childhood. For example, in 2019, the proportion of term babies with a low birth weight, the infant mortality rate, and the prevalence of obesity in children aged 4–5 and 10–11 years in the most disadvantaged areas were more than double the least disadvantaged (PHE 2021a). In addition, almost four times as many five-year-olds in the most disadvantaged areas had dental decay in 2018–2019 when compared to the least disadvantaged (PHE 2021a).

Socioeconomic situation is also associated with vision outcomes. A systematic review by Lane et al. (2018) evaluated whether there is an association between multiple aspects of deprivation and ocular health worldwide. There was much evidence that worse vision outcomes are correlated with ‘multiple deprivation’ (a term that refers to several types of deprivation occurring at once). They also highlight the bidirectional relationship between deprivation and impaired vision, such that there are deprivation-related obstacles to good ocular health, but impaired vision, in turn, presents challenges that can ‘trap’ individuals in deprivation. Evidence is presented for deprivation-related barriers to good ocular health, including poor nutrition; earlier onset of disease but later presentation to services; reduced awareness of disease and participation in screening; access issues and reduced ability to pay for services; reduced adherence; and reduced availability of services.

Within orthoptics, clinicians recognise this relationship between socioeconomic situation and treatment outcome. At a regional meeting of orthoptists in the UK (The British and Irish Orthoptic Society Northern Branch meeting) in June 2022, the authors asked attendees via an anonymous online survey whether they thought there was a relationship between these two factors in their clinic. 100% of the 30 respondents answered ‘yes’. When asked what they had noticed, approximately half of the 41 responses mentioned a problem with attendance at clinics. Other observations from attendees referred to: living conditions; compliance; understanding/engagement; breaking or losing glasses/eye patches; transport issues; cost of attending/glasses; safeguarding issues; language barriers; multiple carers for children; and co-morbidities.

Despite the anecdotal association between socioeconomic circumstances and orthoptic treatment outcomes, no literature review was found to address this issue. Neither was a UK-specific literature review found to address the same issue for ophthalmology as a whole. This is an issue that could be different in other countries due to differing health systems and economic situations, so it is relevant to have a review that is specific to the UK. For orthoptists, ophthalmology is as relevant as orthoptics itself, particularly now that many orthoptists undertake historically ophthalmic roles (Greenwood et al. 2021).

Determining whether there is an association between socioeconomic situation and treatment outcome in orthoptics and ophthalmology is also important ethically because it could reveal health inequality. Furthermore, it is important financially if healthcare appointments are being missed (NHSE 2019), which there is evidence for in the wider National Health Service (Wilson & Winnard 2022). The potential financial and ethical issues are closely related because missed appointments that must be rearranged will also increase waiting lists for future appointments, which in turn delays the available treatment for all patients waiting for appointments (NHSE 2014).

Therefore, the aim of this literature review is to evaluate evidence for an association between socioeconomic situation and ophthalmic or orthoptic conditions and their treatment outcomes within the UK. The section addressing ophthalmology will cover visual acuity and refractive error, then focus on prevalent eye diseases in the UK: cataract, glaucoma, age-related macular degeneration, and diabetic retinopathy (FfS 2019). The section addressing orthoptics will cover amblyopia and strabismus because these are the orthoptic conditions for which relevant literature is available.

## Methods

Keyword searches were conducted on Google Scholar and the University of Liverpool library catalogue (University of Liverpool n.d.), which includes access to over 500 different databases. Search terms included ‘amblyopia’, ‘treatment outcome’, ‘socioeconomic’, ‘social deprivation’, ‘deprivation’, ‘compliance’, ‘vision’, ‘visual’, ‘refract’, ‘cataract’, ‘glaucoma’, ‘age-related macular degeneration’, ‘diabetic retinopathy’, ‘ophthalmology’, ‘orthoptic’, ‘strabismus’, ‘diplopia’, ‘binocular’, and ‘ocular motility’. Search results were screened by title, then by abstract if they appeared relevant, and then by full article if they still appeared relevant. Additional articles were also identified from reference lists, and forward citation searches were performed on key articles. Results for the main analyses were limited to full papers from this century, specific to the UK, written in English. Literature was only included from pre-2000 if more recent evidence was insufficient.

## Socioeconomic Situation and Ophthalmology

Details of all studies discussed in this section are included in Table 2.

**Table 2:** Studies reporting on socioeconomic situation (SES) and ophthalmology.


OPHTHALMIC CONDITION		REFERENCE	MEASURE OF SES	RELATIONSHIP WITH SES (OR = ODDS RATIO)	DATA COLLECTION METHOD	SAMPLE SIZE

**Visual acuity**	VA 0.1-1.3 logMAR	Dawes et al 2014	Townsend index	Higher incidence with disadvantage (OR 1.5–2.0, p < 0.001).	Examinations, questionnaires.	116682

	VA >0.3	McKibbin et al 2018	Townsend index	More likely with disadvantage (relative risk ratio 1.25, 1.84 for monocular and binocular impairment, p < 0.05 for both).	Examinations, participant-reported eye history.	32797

	VA 0.3 or worse. Near VA N8 or worse. <2 images seen on Lang stereotest.	Rahi et al 2009	Occupation; accommodation in childhood; father’s occupation at their birth.	More likely with disadvantage (OR 1.06–3.28).	Examinations, interviews.	9330

	VA 0.3 or worse	Yip et al 2013	IMD	More likely in disadvantaged areas (OR 1.7, 95% CI 1.1–2.6, p = 0.03).	Examinations, questionnaires.	8467

**Refractive error**	Refractive error	Foster et al 2010	Finishing school; occupation.	0.6D more hypermetropic if do not finish school (p < 0.0001). No association with occupational class.	Examinations, questionnaires.	2210

	Refractive error	Goverdhan et al 2011	IMD	0.24mm shorter axial length and 0.12 dioptres (D) greater astigmatism if disadvantaged, no association with spherical refraction.	Database.	7652

	Myopia	Rahi et al 2011	Occupation; education level.	Myopia at <16 years old associated with paternal non-manual occupations (OR 1.8, p < 0.05). Myopia (but not mild/moderate or high myopes analysed separately) associated with non-manual occupations (OR 1.2, p < 0.05). Myopia (but not myopia ≥6D) associated with education above A levels (OR 1.4, p < 0.05).	Examinations, interviews.	2487

	Uncorrected refractive error	Sherwin et al 2012	Education level; Registrar General’s occupation-based system.	No association	Examinations, questionnaires.	4275

**Cataract**	VA ≤0.3 at cataract pre-assessment	Chua et al 2013	SIMD	Less likely if disadvantaged (p = 0.03).	Case note review.	184

	Cataract surg. wait	Cooper et al 2009	Carstairs index	10 days longer in 1997 (p < 0.001), 10 days shorter in 2007 (p < 0.001), if disadvantaged.	National hospital activity data.	2568318

	Pre-op cataract VA	Goyal et al 2004	WIMD	Worse in disadvantaged areas (0.85 vs 0.76, visual function index of 66 vs 72).	VA measure, questionnaires.	112

	Pre-op cataract VA	Johnston et al 2020	IMD	Worse if disadvantaged (0.6, 0.5 for others; 23.6% with ≥1.0 logMAR from most disadvantaged, 12.5% from least).	Medical records.	154223

	Rate of cataract surgery	Keenan et al 2007	IMD	Higher if disadvantaged (r^2^ = 0.24).	Hospital inpatient enquiry, hospital episode statistics, Oxford record linkage study.	All cataract surgery 1960s-2003.

**Glaucoma**	Glaucoma	Fraser et al 2001	Jarman’s score; occupation; housing; car access.	More advanced glaucoma at presentation if disadvantaged (OR 1.01–69.2).	Prospective case control study.	220

	Advanced open-angle glaucoma	King et al 2023	IMD	Younger age at diagnosis if disadvantaged (mean 62 years for least advantaged; 70 for most advantaged, no stats reported). Visual field (VF) worse at baseline if disadvantaged (mean deviation (MD) -17, vs -13 in most advantaged group, Pearson correlation between IMD and VF MD 0.27 and 0.23 for each eye, p < 0.001 for each eye). No association with treatment effect.	Multicentre RCT for different treatments.	450

	Angle closure glaucoma	Nessim et al 2009	Townsend index; IMD.	More likely if disadvantaged (p < 0.001).	Hospital records.	139

	Glaucoma	Ng et al 2010	SIMD	More advanced at presentation if disadvantaged (severe in 45% of most deprived ranks and 10% of least deprived ranks).	Case note review.	122

	Glaucoma	Rathore et al 2023	IMD	Advanced visual field loss at presentation more likely if disadvantaged (OR1.41, p < 0.001 in least advantaged decile, OR0.75, p < 0.001 in most, both compared to 5^th^ decile). No association with rapid visual field progression.	Electronic medical records.	44956 at presentation, 15094 for longitudinal data.

	Urgent referral with narrow angles.	Saxby et al 2022	SIMD	More likely to be disadvantaged (SIMD deciles 5&6) than routine referrals (deciles 6&7) (p = 0.033, 0.025, <0.001, <0.01).	Case note review.	718

	Glaucoma	Shweikh et al 2015	Townsend index	Greater incidence if disadvantaged (Townsend –0.72 for glaucoma, –0.95 without glaucoma, p < 0.001).	Questionnaires.	112690

	Glaucoma	Sukumar et al 2009	Acorn index	Worse visual field at presentation if disadvantaged (correlation –0.19, p = 0.04).	Database.	113

	New glaucoma suspects	Wong et al 2023	SIMD	Worse visual field at presentation if disadvantaged (visual field mean deviation decreased by 0.038dB with each 100-point lower SIMD, R^2^ = 0.0257, p = 0.002).	Medical records.	472

**AMD**	Wet AMD	More et al 2019	IMD	More severe at presentation if disadvantaged (OR 4.07, 95% CI 1.5–11.0, p = 0.006). No association with treatment outcome.	Case note review	524

	AMD	Relton et al 2022a	IMD	VA at treatment initiation 0.09 logMAR worse if disadvantaged.	Medical records.	9116

	AMD	Relton et al 2022b	IMD	VA 0.029 worse after 12 months of treatment, if disadvantaged.	Medical records.	7686

	Neovascular AMD	Sharma et at 2014	IMD	No association with: time to first appointment; DNA rate; cancellation rate; time between presentation and first treatment; a patient not receiving treatment; the number of treatments received prior to registration; the level of VA at which registration occurred. Earlier CVI registration (–0.246, p = 0.007) and worse presenting VA (0.185, p = 0.013 for study eye and 0.225, p = 0.013 for better eye) if disadvantaged.	Medical records.	120

	AMD	Vassilev et al 2015	Townsend index	No association	Medical research database.	29905

	AMD	Yip et al 2015	IMD	More likely if disadvantaged (OR 0.56, 95% CI 0.36-0.89, p = 0.02).	Examinations, questionnaires.	5182

	AMD	Yip et al 2021	Education level; employment; household income; Townsend index.	Reduced odds of AMD without qualifications (OR 1.16&1.30 for lower&higher quals). 24% greater risk of AMD if disadvantaged. Townsend scores not associated with AMD.	Questionnaires.	133339

**Diabetic retinopathy**	Diabetic retinopathy	Gulliford et al 2010	IMD	Reduced attendance at screening if disadvantaged (23% vs 21%, adjusted OR 1.37, p < 0.001). No association with sight threatening retinopathy incidence.	Medical records.	31484

	Diabetic retinopathy	Hipwell et al 2014	Homelessness; employment.	Being in temporary accommodation meant one patient could not access screening. Working patients forgot to organise screening appointments.	Qualitative study with interviews.	62

	Diabetic retinopathy	Kliner et al 2012	IMD	Increased prevalence if disadvantaged (*r* = 0.634, *P* < 0.0001). Reduced screening uptake if disadvantaged (no values). Increased referral from screening if disadvantaged (*r* = 0.728, *P* < 0.0001).	Records.	All available records for Bradford&Airedale GP patients 2009–2010.

	Diabetic retinopathy	Lane et al 2015	IMD	Presentation with advanced disease associated with more disadvantage (p < 0.001). No association with time to treatment (R3 disease) or non-attendance.	Records review.	100

	Diabetic retinopathy	Lawrenson et al 2020	IMD	Reduced attendance at screening if disadvantaged (OR 0.73). At screening, more retinopathy requiring surveillance/referral if disadvantaged (OR 1.42).	Records.	Cohort A = 97048. Cohort B = 291296.

	Diabetic retinopathy	Leese et al 2008	Carstairs score	Most disadvantaged were 2.32 times more likely to miss screening appointments.	Databases, records.	15150

	Diabetic retinopathy	Low et al 2015	SIMD	Increased prevalence for type 1 diabetes (OR 2.4, 95% CI 1.36 to 4.27, p = 0.002), but not for type 2, if disadvantaged.	Database.	20058

	Diabetic retinopathy	Mathur et al 2017	IMD	No linear relationship.	Database.	7707475

	Diabetic retinopathy	Millett and Dodhia 2006	IMD	Reduced attendance at screening if disadvantaged (OR 0.58). No association with prevalence of retinopathy at screening.	Disease register.	8066

	Diabetic retinopathy	Moreton et al 2017	IMD	Less likely to attend screening if disadvantaged (OR 0.75, p = 0.02).	Database.	21797

	Diabetic retinopathy	Orton et al 2013	IMD	Reduced attendance at screening if disadvantaged (OR 1.19, p < 0.001).	Records.	47111

	Diabetic retinopathy	Scanlon et al 2008	IMD	Increased prevalence (OR 0.98, 95% CI 0.95-1.02) if disadvantaged.	Database.	10312

	Diabetic retinopathy	Shah et al 2021a	IMD	If no retinopathy at screening, more likely to be advantaged (p < 0.001). Those with retinopathy at screening less likely to be advantaged (22% vs expected 25%). Less likely to develop retinopathy in 7 years after diabetes diagnosis if advantaged (OR0.63, p < 0.001). No association with progression of retinopathy.	Database.	11399

	Diabetic retinopathy	Waqar et al 2012	IMD; housing and lifestyle.	Reduced attendance at screening if disadvantaged (11 vs 6% for 1st missed appt, 3 vs 1% for 2nd).	Records.	2137


### Reduced visual acuity (VA)

Within the UK, several studies with adult participants have reported a higher incidence of visual impairment in socioeconomically disadvantaged individuals. Rahi, Cumberland & Peckham (2009) reported that individuals with unskilled manual occupations, a history of more crowded accommodation as children, and fathers with manual occupations at their birth were more likely (odds ratios of 1.23–2.55, 1.35–3.28, 1.06–1.47, respectively) to have impaired vision (distance VA of 0.3 logMAR or worse, near VA worse than N8, or stereovision of less than two images seen on a Lang test). Dawes et al. (2014) also found a higher incidence (odds ratio 1.5–2.0, p < 0.001) of visual impairment (classed as between 0.1 and 1.3 logMAR) in individuals with socioeconomic disadvantage. It could be argued that their classification of visual impairment is too sensitive, though, because a VA of 0.12 logMAR for example, would be classified as visually impaired, and this level of VA would not usually be problematic for patients; they would be legal to drive (GDS 2012) and would pass school vision screening assessments (UKNSC 2023). Yip et al. (2013) also found that people with reduced visual acuity (0.3 logMAR or worse) were more likely to live in the most disadvantaged areas, even after adjusting for previous cataract surgery and markers of individual socioeconomic situation (odds ratio 1.7, 95% confidence interval 1.1–2.6, p = 0.03). It was reported that the effect was partly due to uncorrected refractive error, and it was concluded that targeting uncorrected refractive error in disadvantaged areas may address the inequality. It is of note that refractive error in this study was identified by VA improving with a pinhole, not by refraction. However, a pinhole test would not identify all cases of uncorrected refractive error or be specific to this cause, so this explanation should be interpreted cautiously. Indeed, Sherwin et al. (2012) found uncorrected refractive error not to be associated with educational background or ‘social class’. They did, however, also define uncorrected refractive error as presenting visual acuity worse than 0.3 logMAR, which improved with a pinhole. This, again, is not ideal for the same sensitivity and specificity reasons described above. While the explanation by Yip et al. (2013) is debatable, McKibbin, Farragher & Shickle (2018) report similar associations between visual acuity and socioeconomic situation. They found that 40- to 69-year olds living in the most disadvantaged areas were 25% more likely to have monocular visual impairment (VA worse than 0.3 logMAR) compared to those living in the most advantaged areas. Additionally, this percentage increased to 84% for binocular visual impairment. It is worth noting that the studies comparing visual acuity and socioeconomic situation described above do not adjust for coexisting eye disease (apart from previous cataract surgery and uncorrected refractive error by Yip et al. (2013)), so there may be some overlap with associations described in the following sections of this article.

### Refractive error

Goverdhan et al. (2011) found that socioeconomic disadvantage was associated with shorter axial length (0.24mm difference between the highest and lowest IMD quintiles) and greater astigmatism (0.12 dioptres (D) difference between the highest and lowest quintiles), but not with spherical refraction. The absence of an association with spherical refraction and the level of difference in astigmatism would suggest that there is no clinically significant association here. Foster et al. (2010) found that ‘occupational class’ had no association with refractive error, and Rahi, Cumberland & Peckham (2011) found that myopia was significantly associated with non-manual occupations, but only when all their myopes were grouped together. When mild/moderate or high myopes were analysed separately, no significant association was found. A higher education level had a more convincing association with myopia (Foster et al. 2010; Rahi, Cumberland & Peckham 2011).

### Cataract

For patients listed for cataract surgery, several authors have reported worse-presenting visual acuity in individuals who are socioeconomically disadvantaged (Chua et al. 2013; Goyal, Shankar & Sullivan 2004; Johnston et al. 2020). Only the results from Chua et al. (2013) were statistically analysed. They report that 69% of patients had VA of 6/12 or better, which was found to be associated with affluence (p = 0.03). Other evidence relating to cataracts was sparse: Keenan et al. (2007) found a correlation between socioeconomic disadvantage and a higher annual rate of cataract surgery (rates not stated, but there was an overall range of 172 to 548 per 100,000); and Cooper et al. (2009) found that by 2007, there was an approximately 10-day shorter wait for cataract surgery for the most socioeconomically disadvantaged individuals, compared to the most advantaged. Evidence was not found to explain whether the last two findings were due to a greater need amongst the socioeconomically disadvantaged (more cataracts or being prioritised due to worse-presenting VA, for example) or greater access with the same level of need.

### Glaucoma

There is also evidence of an association between socioeconomic disadvantage and glaucoma. 1,916 of 112,690 people in the UK Biobank study reported a glaucoma diagnosis, and those who reported the diagnosis had a greater deprivation score (–0.72 Townsend score) than those who did not report it (–0.95 Townsend score) (p < 0.001) (Shweikh et al. 2015). For acute primary angle closure glaucoma, Nessim et al. (2009) found that 139 consecutive patients presenting with angle closure were more likely (p < 0.001) to come from areas with a high level of deprivation. Saxby et al. (2022) also reported that 718 consecutive patients referred for laser iridotomy for narrow anterior chamber angles were more likely to be socioeconomically disadvantaged (Scottish Index of Multiple Deprivation (SIMD) deciles 5 and 6 for two centres) if they were referred urgently with acute primary angle closure, compared to patients referred routinely (SIMD deciles 6 and 7). They did not, however, include anyone who was not referred for laser iridotomy. It seems reasonable to assume that this would not miss many individuals with acute angle closure because the symptoms would usually be significant enough for people to present, but there are likely to be individuals with asymptomatic narrow angles in the general population that have not been identified by eye care services. Other evidence suggests that this asymptomatic group who have not presented to eye care services, which would be included in the routine referrals, could include a greater proportion who are socioeconomically underserved (Dickey et al. 2012; Majeed et al. 2008). Therefore, it is possible that the results of this last study are skewed.

There is significant evidence that socioeconomic disadvantage can delay presentation in glaucoma. Ng et al. (2010) found that new glaucoma patients were more likely to present with severe glaucoma (45% incidence) if they had the most deprived SIMD ranks, compared to the least deprived SIMD ranks (10% incidence). Fraser et al. (2001) also found a relationship between socioeconomic disadvantage and advanced glaucoma. They report odds ratios for advanced glaucoma at presentation varying from 1.01 to 69.2 for markers of socioeconomic disadvantage, including the Jarman’s underprivileged area score (Jarman 1983); ‘occupational class’; housing tenure; and access to a car. Sukumar et al. (2009) related the extent of visual field loss for new glaucoma patients to the Acorn socioeconomic index. They also found a correlation (coefficient -0.19) between socioeconomic disadvantage and greater visual field loss at presentation. More recently, King et al. (2023) reported the same association within the group of glaucoma patients with advanced disease at baseline (correlation 0.27 and 0.23 for each eye, p < 0.001 for each). Rathore et al. (2023) also concurred, finding that patients were 7% more likely to have advanced visual field loss at presentation to hospital eye services if they were from the least advantaged IMD decile, compared to the most advantaged (18% versus 11%, respectively, odds ratio 1.41 for the least advantaged, 0.75 for the most advantaged, p < 0.001 for both groups compared to the fifth decile). Wong et al. (2023) also concurred, finding a 0.038 dB reduction in visual field mean deviation value for the worst eye at presentation for each 100-point lower SIMD (R^2^ = 0.0257, p = 0.002).

Rathore et al. (2023) also examined their data longitudinally and calculated the proportion of patients with rapid visual field loss over time in each IMD decile. They found no association between IMD decile and rapid visual field loss, which indicates that once these patients are under the hospital eye service, as long as they attend, there is no apparent socioeconomic inequality in their glaucoma treatment outcome. This conclusion is, however, based upon data from patients who attended the eye clinic at least six times, which could have skewed results as socioeconomically underserved individuals may use eye-care services less (Dickey et al. 2012; Majeed et al. 2008). Indeed, King et al. (2023) report that patients displayed advanced glaucoma at a younger age if they were disadvantaged (mean 62 years, versus 70 years in the most advantaged group, no statistics reported). This could be due to the delay in presentation, because even if visual field loss occurs at the same rate for all patients, those with further-progressed disease at presentation would reach advanced disease sooner. Follow-up results from King et al. (2023) would seem to support this idea because socioeconomic situation had no association with treatment effect at the 24-month visit.

### Age-related macular degeneration (AMD)

Several studies have investigated the likelihood of AMD in relation to socioeconomic situation. Yip et al. (2021) reported on 133,339 participants from the UK Biobank study and found that having no academic qualifications reduced the odds of AMD (odds ratios 1.16 and 1.30), but having an annual household income below £18,000 produced a 24% greater risk of AMD compared to an annual income above £100,000. These two findings seem contradictory, but the authors offer an explanation: data was collected via participant-administered questionnaires, and people with higher levels of education may be more aware of early disease, so AMD in participants without qualifications could be underreported. Vassilev et al. (2015) and Yip et al. (2021) both report no association between socioeconomic situation and the presence of AMD when socioeconomic situation was measured using the Townsend Deprivation Index. When measured by the IMD score, Yip et al. (2015) found a greater chance of AMD (odds ratio 0.56) with socioeconomic disadvantage. Therefore, findings seem to vary depending on the method of measuring socioeconomic disadvantage.

Relton et al. (2022a) and Sharma et al. (2014) report worse-presenting VA for the socioeconomically disadvantaged with AMD in contrast with Acharya et al. (2008), who report no association between socioeconomic situation and presenting VA. Despite the apparently different reports, Relton et al. (2022a) report only 0.09 logMAR worse VA at treatment initiation, which would be considered clinically insignificant. Sharma et al. (2014) report the correlation between socioeconomic disadvantage and lower presenting VA as only weakly positive, with a correlation of 0.185 for the study eye (but still statistically significant, p = 0.013), and their cohort was all patients who were eventually registered as sight impaired or severely sight impaired, which might also explain a difference in findings. More et al. (2019) report a higher incidence (values not stated) of severe AMD at presentation for the socioeconomically disadvantaged when analysed as a category of being able to see less than 35 letters on a standard ETDRS chart (odds ratio 4.07). The median number of letters read on an ETDRS chart binocularly in the ‘most deprived areas’ was 47, compared to 57 in the ‘less deprived areas’, but there was high variability in the most disadvantaged areas, with analysis showing the difference was not statistically significant.

Reassuringly, there is evidence that treatment outcome (More et al. 2019; Relton et al. 2022b) and the service received by AMD patients (Sharma et al. 2014), is not associated with socioeconomic situation.

### Diabetic retinopathy

Twelve studies were found that addressed attendance at diabetic retinopathy screening in the UK. Nine of these found that attendance was lower for socioeconomically disadvantaged individuals (Fraser et al. 2011; Gulliford et al. 2010; Kliner et al. 2012; Lawrenson et al. 2020; Leese et al. 2008; Millett & Dodhia 2006; Moreton et al. 2017; Orton et al. 2013; Waqar et al. 2012). In contrast, Buch et al. (2005) and Lane et al. (2015) found no association with attendance. For Lane et al. (2015), it appears that this was for a mixture of hospital and screening appointments for patients who had attended at least one screening appointment and been referred to a hospital with advanced disease, so this is not the same as just attendance at retinopathy screening. The final study of the 12 by Hipwell et al. (2014) was a qualitative study addressing individual experiences of diabetic retinopathy screening. They report mixed associations between socioeconomic situation and attendance at diabetic retinopathy screening: one patient could not access the screening due to being homeless, but conversely, working people reported problems with forgetting to organise their screening appointments.

Evidence of reduced attendance at diabetic retinopathy screening for disadvantaged individuals could explain findings by Denniston et al. (2019) and Lane et al. (2015) that the presentation of diabetic retinopathy is delayed for these individuals. It could also explain why a greater proportion of screening appointments result in referrals to hospital services for socioeconomically disadvantaged individuals (Kliner et al. 2012; Lawrenson et al. 2020).

There was also evidence of an increased prevalence of diabetic retinopathy for individuals who are socioeconomically disadvantaged (Kliner et al. 2012; Shah et al. 2021a). Conversely, other authors (Mathur et al. 2017; Millett & Dodhia 2006; Scanlon et al. 2008) found no relationship with the prevalence of diabetic retinopathy at screening. Low et al. (2015) found an increased prevalence of diabetic retinopathy at screening for type 1 diabetes but not for type 2. For most of the studies that did not find an increased prevalence of all types of diabetic retinopathy, their data was collected from routine screening appointments (for Shah et al. 2021a, it was at screening only for newly diagnosed diabetics). This method would not include all cases of diabetic retinopathy because those that were being seen by the hospital eye service (with the most advanced disease) would not be included in screening. Therefore, this could have skewed the findings. Further clarity in this area is required.

### Summary

In the UK, there is robust evidence that the following are associated with socioeconomic disadvantage: reduced visual acuity; reduced attendance at diabetic retinopathy screening appointments; and delayed presentation of glaucoma, cataracts, and diabetic retinopathy. However, the evidence linking an increase in glaucoma with socioeconomic disadvantage and the association with AMD is mixed, and more clarity is needed in these areas.

## Socioeconomic Situation and Orthoptics

Details of studies supporting conclusions in this section are included in Table 3.

**Table 3:** Studies reporting on socioeconomic situation (SES) and orthoptics.


CONDITION		REFERENCE	MEASURE OF SES	RELATIONSHIP WITH SES (OR = ODDS RATIO)	DATA COLLECTION METHOD	SAMPLE SIZE

**Childhood amblyopia, refractive error and strabismus**	Refractive error	Kearney et al 2022	SIMD	Increased spectacle supplement claims if disadvantaged (regression coefficient –2.27, p < 0.001).	Administrative records.	108043

	Childhood esotropia, hypermetropia, amblyopia.	Majeed et al 2008	Standard Occupational Classification of parents; housing tenure; maternal education level.	Hypermetropia more likely if disadvantaged (OR1.69, p = 0.01). No significant association with amblyopia (p = 0.08) or esotropia (p = 0.23). Less likely to see an eye-care specialist if disadvantaged (OR0.83, p = 0.02).	Examinations, questionnaires, records.	8271

	Pre-school vision screening result	O’Colmain et al 2015	SIMD; Health Plan Indicator (HPI) category.	‘Pass’ more likely if advantaged by SIMD (OR1.4, p = 0.017). ‘Fail’ more likely if disadvantaged by HPI (OR3.59, p = 0.001)	Database	1170

	Treatment outcomes following pre-school vision screening	O’Colmain et al 2020	SIMD; Health Plan Indicator (HPI) category.	Poor attendance more likely if disadvantaged (OR2.19, p = 0.003 by SIMD, OR3.94, p = 0.002 by HPI). More likely to be ‘noncompliant’ with treatment if disadvantaged by HPI (OR9.97, p < 0.001). Poor attenders more likely to have residual amblyopia (OR6.42, p < 0.001). Poor attenders more likely to have poor/no BSV at baseline (79%vs70.8%) & discharge (68.5%vs20.1%) (p < 0.001). For good attenders, no association with visual outcome by SIMD. For good attenders who were disadvantaged by HPI, more likely to have final VA worse than 0.2 logMAR (OR5.37, p < 0.001) and poor/no BSV (OR3.41, p < 0.001).	Records review	430

	Children receiving amblyopia therapy	Smith et al 1995	Townsend score	22% more likely to attend all orthoptic appointments if advantaged (p < 0.0001).	Not explicitly stated	961

	Childhood strabismus, hypermetropia, amblyopia.	Williams et al 2008	Standard Occupational Classification of parents.	2% greater prevalence of hypermetropia if disadvantaged (p = 0.01). Over 80% more likely to be hypermetropic if disadvantaged (OR1.82, p = 0.027). No significant association with strabismus or amblyopia.	Questionnaires, examinations.	7538

**Adult strabismus and amblyopia**	Persisting amblyopia in adulthood	Bountziouka et al 2021	Townsend score	More likely if disadvantaged (OR1.47, p < 0.001). Not associated with educational attainment. Only associated with limited working ability (OR1.3, p = 0.005) and housing tenure (OR1.44, p = 0.01) if aged≥60yrs.	Surveys, examinations, medical records.	19231

	Adult strabismus	Durnian et al 2010	IMD	Lower AS-20 score (poorer quality of life) if disadvantaged (r^2^ = –0.3, p = 0.006).	Questionnaires, medical records.	61

	Adult strabismus	Sim et al 2018	IMD	Better surgical success rate based upon AS-20 scores, if disadvantaged (OR1.07, p = 0.04).	Questionnaires, examinations.	87


### Amblyopia, including childhood refractive error and strabismus

O’Colmain et al. (2015) analysed pre-school vision screening assessments, which would identify amblyopia, in Tayside, Scotland. Children were 1.4 times more likely to pass the assessment if they were advantaged (by SIMD score) and three times more likely to fail if they were from homes needing more support from services (measured by the Health Plan Indicator (HPI), a support category assigned to each child by their health visitor).

Some explanation is offered for this by looking at risk factors for amblyopia, such as refractive error and strabismus (Pascual et al. 2014). In 2008, two separate analyses were published using data from the Avon Longitudinal Study of Parents and Children (ALSPAC). Williams et al. (2008) found an 82% greater risk of hypermetropia for disadvantaged children but no significantly greater risk of strabismus or amblyopia. For esotropia and amblyopia, there was a trend towards lower prevalence for advantaged children, but it did not reach statistical significance. Majeed et al. (2008) also report a 69% greater risk of hypermetropia for disadvantaged children (p = 0.01), but no significant association with esotropia (odds ratio 1.46, p = 0.23) or amblyopia (odds ratio 1.52, p = 0.08). Majeed et al. (2008) report that most patients were in social class 2 (the second most advantaged), with only 12.7% in the least advantaged three social groups (out of six groups in total); in addition, a third of mothers had education beyond A-level, and 75% owned their home. This demonstrates how disadvantaged individuals were underrepresented in this study, as highlighted by the authors (Williams et al. 2008), so the results may not fully reflect their situation.

Maternal smoking during pregnancy is another risk factor for amblyopia (Li et al. 2016). Delpisheh et al. (2006) conducted an analysis of surveys completed by parents in Merseyside between 1993 and 2001. It was found that disadvantaged mothers were 23% more likely to smoke during pregnancy than advantaged mothers. More recently, a government report (PHE 2021b) found that the most disadvantaged women were more than six times as likely (19% vs 3%) to be smokers at their pregnancy booking appointment (generally in the first trimester) and 9% more likely to smoke throughout their pregnancy, compared to the most advantaged. Advantaged women who smoked were 39% more likely to stop smoking in early pregnancy and 9% more likely to stop smoking in late pregnancy, compared to disadvantaged women. It was also reported that pregnant women who had never smoked were 1.3 times more likely to be advantaged. Statistical analyses were not included in the government report, but the percentages appear to show a consistent trend.

The above findings go some way to explain the results of O’Colmain et al. (2015), but it is relevant to consider whether the suggested inequality is resolved with orthoptic therapy. In 2020, O’Colmain et al. reported on children who received orthoptic therapy following pre-school vision screening. Children from more disadvantaged backgrounds (by SIMD) and those from families requiring more support (by HPI category, as described above), were more likely (twice and almost four times as likely, respectively) to have poor attendance at hospital appointments. Poor attendance increased the chances of having residual amblyopia (odds ratio 6.42) and poor or no binocular vision at discharge (49% more likely). For children who attended well, the SIMD score did not affect the overall vision outcome, but those requiring more support at home were still more likely to have worse vision (odds ratio 5.37) and binocularity (odds ratio 3.41) outcomes than their more advantaged peers. This association is also reported to persist into adulthood (Bountziouka, Cumberland & Rahi 2021).

To consider an explanation for the association between amblyopia treatment outcomes and socioeconomic disadvantage, one key factor that affects outcomes is how much treatment is administered by patients and their parents or guardians (Simons & Preslan 1999). O’Colmain et al. (2020) found that the disadvantaged children by HPI category were almost 10 times more likely to be recorded as ‘non-compliant’ with glasses or occlusion. Smith et al. (1995) also measured ‘compliance’ to amblyopia treatment by attendance rates at seven participating English orthoptic clinics and found that attendance in the most advantaged areas was 22% better than in the most disadvantaged areas. In addition, Majeed et al. (2008) found that children from more disadvantaged backgrounds used eye-care services less (odds ratio 0.83). However, Kearney et al. (2022) reported that socioeconomically underserved children were not disadvantaged in accessing NHS spectacles; there were more spectacle supplement claims for disadvantaged children than their advantaged peers. However, the number of spectacle claims would not differentiate between the prevalence of refractive error and service uptake in each socioeconomic group. For example, the most disadvantaged group could have the highest number of spectacle claims, but this could only be 50% of its refractive errors (50% service uptake), and the most advantaged group could have fewer spectacle claims, but this could be 100% of its refractive errors (100% service uptake). Therefore, the results from this final study should be interpreted cautiously.

Overall, limited evidence of reduced amblyopia treatment outcomes for disadvantaged children appears to be partly due to reduced contact with and concordance with eye-care services.

### Adult strabismus

For adults with strabismus, two studies were found that addressed quality of life in relation to socioeconomic situation. Durnian et al. (2010) found that disadvantaged individuals scored lower (poorer quality of life) on the AS-20 strabismus quality of life questionnaire (r^2^ = –0.3). Sim et al. (2018) also reported lower pre-operative AS-20 scores for disadvantaged individuals and marginally (odds ratio 1.07, p = 0.04) more improvement in their score following strabismus surgery, all in comparison to advantaged individuals. The same relationship between socioeconomic situation and quality of life has been reported in other areas of health (Schneider et al. 2022; Shah, Stokes & Sutton 2021b) and one study links this to increased levels of anxiety and depression (Shah, Stokes & Sutton 2021b). For the improvement in score with strabismus surgery, Sim et al. (2018) suggest that the lower pre-operative quality of life scores for disadvantaged individuals may leave more room for improvement. No other relevant evidence was found in this area.

### Summary

In summary, there is limited evidence of the increased prevalence of amblyopia and subsequent barriers to its treatment for socioeconomically underserved children in the UK. There is also evidence of a reduction in quality of life for socioeconomically underserved adults with strabismus, but the literature is very limited within the orthoptic area, and further research is warranted. Further research should particularly explore whether there is a socioeconomic association with the prevalence and treatment outcomes of orthoptic conditions, so that patients from all socioeconomic situations have fair access to treatment and successful outcomes.

## Conclusions

There is limited evidence of amblyopia, visual impairment, and glaucoma being more prevalent in socioeconomically underserved individuals. There is also evidence of barriers to orthoptic and ophthalmic treatment for the same group, such as delayed presentation, reduced attendance at eye-care appointments, and reduced concordance with therapy. These findings suggest health inequalities within ophthalmology and orthoptics, so research is warranted to explore the reasons behind them and identify methods of reducing them.

## References

[1] Acharya, N, et al. 2008. Socio-economic deprivation and visual acuity at presentation in exudative age-related macular degeneration. British Journal of Ophthalmology, 93(5): 627–629. DOI: 10.1136/bjo.2008.147231 19091850

[2] Bountziouka, V, Cumberland, PM and Rahi, JS. 2021. Impact of persisting amblyopia on socioeconomic, health, and well-being outcomes in adult life: Findings from the UK biobank. Value in Health, 24(11): 1603–1611. DOI: 10.1016/j.jval.2021.05.010 34711360

[3] Buch, HN, et al. 2005. An assessment of the coverage of a district-wide diabetic retinopathy screening service. Diabetic Medicine, 22(7): 840–841. DOI: 10.1111/j.1464-5491.2005.01519.x 15975096

[4] Chua, PY, et al. 2013. Relationship between socioeconomic deprivation or urban/rural residence and visual acuity before cataract surgery in northern Scotland. European Journal of Ophthalmology, 23(6): 831–835. DOI: 10.5301/ejo.5000302 23661537

[5] Cooper, ZN, et al. 2009. Equity, waiting times, and NHS reforms: Retrospective study. BMJ, 339(7722): b3264–b3264. DOI: 10.1136/bmj.b3264 19729415 PMC2737605

[6] Dawes, P, et al. 2014. Vision impairment and dual sensory problems in middle age. Ophthalmic & physiological optics: the journal of the British College of Ophthalmic Opticians (Optometrists), 34(4): 479–488. DOI: 10.1177/1367493506062553 24888710 PMC4273649

[7] Delpisheh, A, et al. 2006. Socio-economic status, smoking during pregnancy and birth outcomes: An analysis of cross-sectional community studies in Liverpool (1993–2001). Journal of Child Health Care, 10(2): 140–148. DOI: 10.1177/1367493506062553 16707542

[8] Denniston, AK, et al. 2019. United Kingdom Diabetic Retinopathy Electronic Medical Record (UK DR EMR) users group: Report 4, real-world data on the impact of deprivation on the presentation of diabetic eye disease at hospital services. British Journal of Ophthalmology, 103(6): 837–843. DOI: 10.1136/bjophthalmol-2018-312568 30269098 PMC6582816

[9] Dickey, H, et al. 2012. Utilisation of eye-care services: The effect of Scotland’s free eye examination policy. Health Policy, 108(2–3): 286–293. DOI: 10.1016/j.healthpol.2012.09.006 23063565

[10] Durnian, JM, et al. 2010. The psychosocial effects of strabismus: Effect of patient demographics on the AS-20 score. Journal of American Association for Pediatric Ophthalmology and Strabismus, 14(6): 469–471. DOI: 10.1016/j.jaapos.2010.08.013 21168068

[11] Dymond-Green, N. 2020. How can we calculate levels of deprivation or poverty in the UK? (part 1), 24 June 2020. Available at http://blog.ukdataservice.ac.uk/deprived-or-live-in-poverty-1/ [Last accessed 30 November 2020].

[12] Fight for Sight (FfS). 2019. Facts about sight loss. Available at https://www.fightforsight.org.uk/about-the-eye/facts-about-sight-loss/ [Last accessed 10 January 2024].

[13] Foster, PJ, et al. 2010. Refractive error, axial length and anterior chamber depth of the eye in British adults: the EPIC-Norfolk Eye Study. British Journal of Ophthalmology, 94(7): 827–830. DOI: 10.1136/bjo.2009.163899 20606021

[14] Fraser, S, et al. 2001. Deprivation and late presentation of glaucoma: Case-control study. BMJ: British Medical Journal, 322(7287): 639–643. DOI: 10.1136/bmj.322.7287.639 11250847 PMC26542

[15] Fraser, S, et al. 2011. Sociodemographic differences in diabetic retinopathy screening; using patient-level primary care data for health equity audit. Clinical Audit, 3: 7–15. DOI: 10.2147/CA.S25313

[16] Goverdhan, S, et al. 2011. Shorter axial length and increased astigmatic refractive error are associated with socio-economic deprivation in an adult UK cohort. Ophthalmic Epidemiology, 18(1): 44–47. DOI: 10.3109/09286586.2010.528853 21091202

[17] Government Digital Service (GDS). 2012. Driving eyesight rules. Available at https://www.gov.uk/driving-eyesight-rules [Last accessed 10 January 2024].

[18] Goyal, R, Shankar, J and Sullivan, S. 2004. Referrals for cataract surgery: Variations between different geographic areas within a Welsh Health Authority. Eye, 18(8): 773–777. DOI: 10.1038/sj.eye.6700724 14716335

[19] Greenwood, V, et al. 2021. Changing practice for the non-medical ophthalmic hospital workforce in the UK—a snapshot survey. Eye, 35(7): 1886–1894. DOI: 10.1038/s41433-020-0955-4 32884134 PMC8225866

[20] Gulliford, MC, et al. 2010. Socio-economic and ethnic inequalities in diabetes retinal screening. Diabetic Medicine, 27(3): 282–288. DOI: 10.1111/j.1464-5491.2010.02946.x 20536490

[21] Hipwell, AE, et al. 2014. Attitudes, access and anguish: a qualitative interview study of staff and patients’ experiences of diabetic retinopathy screening. BMJ Open, 4(12): e005498. DOI: 10.1136/bmjopen-2014-005498 PMC4267079 25510885

[22] Information Analysis Directorate (IAD). 2021. Life expectancy in Northern Ireland 2018–20, December 2021. Available at https://www.health-ni.gov.uk/sites/default/files/publications/health/hscims-life-expectancy-ni-2018-20.pdf [Last accessed 3 August 2022].

[23] Jarman, B. 1983. Identification of underprivileged areas. British Medical Journal (Clinical Research Ed.), 286(6379): 1705–1709. DOI: 10.1136/bmj.286.6379.1705 6405943 PMC1548179

[24] Johnston, RL, et al. 2020. The Royal College of Ophthalmologists’ National Ophthalmology Database study of cataract surgery: Report 4, equity of access to cataract surgery. Eye, 34(3): 530–536. DOI: 10.1038/s41433-019-0524-x 31358923 PMC7042319

[25] Kearney, S, et al. 2022. Socio-economic differences in accessing NHS spectacles amongst children with differing refractive errors living in Scotland. Eye, 36(4): 773–780. DOI: 10.1038/s41433-021-01536-8 33875827 PMC8956614

[26] Keenan, T, et al. 2007. Time trends and geographical variation in cataract surgery rates in England: Study of surgical workload. British Journal of Ophthalmology, 91(7): 901–904. DOI: 10.1136/bjo.2006.108977 17540694 PMC1955650

[27] King, AJ, et al. 2023. Effects of socioeconomic status on baseline values and outcomes at 24 months in the Treatment of Advanced Glaucoma Study randomised controlled trial. British Journal of Ophthalmology, p.bjophthalmol-2022-321922. DOI: 10.1136/bjo-2022-321922 36596663

[28] Kliner, M, et al. 2012. Diabetic retinopathy equity profile in a multi-ethnic, deprived population in northern England. Eye, 26(5): 671–677. DOI: 10.1038/eye.2012.3 22302063 PMC3351065

[29] Lamnisos, D, Lambrianidou, G and Middleton, N. 2019. Small-area socioeconomic deprivation indices in Cyprus: Development and association with premature mortality. BMC Public Health, 19(1): 627. DOI: 10.1186/s12889-019-6973-0 31118020 PMC6532164

[30] Lane, M, et al. 2015. Social deprivation as a risk factor for late presentation of proliferative diabetic retinopathy. Clinical Ophthalmology, 9: 347–352. DOI: 10.2147/OPTH.S73272 25733801 PMC4337620

[31] Lane, M, et al. 2018. Multiple deprivation, vision loss, and ophthalmic disease in adults: Global perspectives. Survey of Ophthalmology, 63(3): 406–436. DOI: 10.1016/j.survophthal.2017.10.009 29100897

[32] Lawrenson, JG, et al. 2020. Trends in diabetic retinopathy screening attendance and associations with vision impairment attributable to diabetes in a large nationwide cohort. Diabetic Medicine, 38(4): e14425. DOI: 10.1111/dme.14425 33064854 PMC8048647

[33] Leese, GP, et al. 2008. Screening uptake in a well-established diabetic retinopathy screening program: The role of geographical access and deprivation. Diabetes Care, 31(11): 2131–2135. DOI: 10.2337/dc08-1098 18728235 PMC2571062

[34] Li, L, et al. 2016. A meta-analysis for association of maternal smoking with childhood refractive error and amblyopia. Journal of Ophthalmology, 2016: 1–7. DOI: 10.1155/2016/8263832 PMC4876230 27247800

[35] Low, L, et al. 2015. Impact of socioeconomic deprivation on the development of diabetic retinopathy: A population-based, cross-sectional and longitudinal study over 12 years. BMJ Open, 5(4): e007290–e007290. DOI: 10.1136/bmjopen-2014-007290 PMC4401835 25877277

[36] Majeed, M, et al. 2008. Are there inequities in the utilisation of childhood eye-care services in relation to socio-economic status? Evidence from the ALSPAC cohort. British Journal of Ophthalmology, 92(7): 965–969. DOI: 10.1136/bjo.2007.134841 18480307

[37] Marmot, M. 2010. Fair society, healthy lives, February 2010. Available at https://www.instituteofhealthequity.org/resources-reports/fair-society-healthy-lives-the-marmot-review/fair-society-healthy-lives-full-report-pdf.pdf [Last accessed 22 February 2022].

[38] Martin, D. 2007. Townsend deprivation index. Available at https://www.restore.ac.uk/geo-refer/36229dtuks00y19810000.php [Last accessed 30 November 2020].

[39] Mathur, R, et al. 2017. Population trends in the 10-year incidence and prevalence of diabetic retinopathy in the UK: A cohort study in the Clinical Practice Research Datalink 2004–2014. BMJ Open, 7(2): e014444. DOI: 10.1136/bmjopen-2016-014444 PMC5337737 28246144

[40] McKibbin, M, Farragher, TM and Shickle, D. 2018. Monocular and binocular visual impairment in the UK Biobank study: prevalence, associations and diagnoses. BMJ Open Ophthalmology, 3(1): e000076. DOI: 10.1136/bmjophth-2017-000076 PMC5895967 29657974

[41] Millett, C and Dodhia, H. 2006. Diabetes retinopathy screening: Audit of equity in participation and selected outcomes in south east London. Journal of Medical Screening, 13(3): 152–155. DOI: 10.1258/096914106778440608 17007657

[42] Ministry of Housing, Communities and Local Government (MHCLG). 2019. Statistical Release, September 2019. Available at https://assets.publishing.service.gov.uk/government/uploads/system/uploads/attachment_data/file/835115/IoD2019_Statistical_Release.pdf [Last accessed 12 November 2020].

[43] More, P, et al. 2019. Socio-economic status and outcomes for patients with age-related macular degeneration. Eye, 33(8): 1224–1231. DOI: 10.1038/s41433-019-0393-3 30858565 PMC7005865

[44] Moreton, RBR, et al. 2017. Factors determining uptake of diabetic retinopathy screening in Oxfordshire. Diabetic Medicine, 34(7): 993–999. DOI: 10.1111/dme.13350 28295529 PMC5485053

[45] National Records of Scotland (NRS). 2022. Healthy life expectancy 2018–2020. Available at https://www.nrscotland.gov.uk/statistics-and-data/statistics/statistics-by-theme/life-expectancy/healthy-life-expectancy-in-scotland/2018-2020#:~:text=In%20Scotland%20between%202018%2D2020 [Last accessed 3 August 2022].

[46] Nessim, M, et al. 2009. Research into Glaucoma And Ethnicity (ReGAE) 8: Is there a relationship between social deprivation and acute primary angle closure? British Journal of Ophthalmology, 94(10): 1304–1306. DOI: 10.1136/bjo.2009.160721 19767334

[47] Ng, WS, et al. 2010. The effect of socio-economic deprivation on severity of glaucoma at presentation. British Journal of Ophthalmology, 94(1): 85–87. DOI: 10.1136/bjo.2008.153312 19628488

[48] NHS England (NHSE). 2014. NHS England using technology to beat cost of missed appointments, 5 March 2014. Available at https://www.england.nhs.uk/2014/03/missed-appts/ [Last accessed 10 January 2024].

[49] NHS England. 2019. Missed GP appointments costing NHS millions, 2 January 2019. Available at https://www.england.nhs.uk/2019/01/missed-gp-appointments-costing-nhs-millions/ [Last accessed 10 January 2024].

[50] Northern Ireland Statistics and Research Agency (NISRA). 2017. Northern Ireland Multiple Deprivation. Available at https://www.nisra.gov.uk/sites/nisra.gov.uk/files/publications/NIMDM17-%20with%20ns.pdf [Last accessed 10 January 2024].

[51] O’Colmain, U, et al. 2015. Vision screening in children: a retrospective study of social and demographic factors with regards to visual outcomes. British Journal of Ophthalmology, 100(8): 1109–1113. DOI: 10.1136/bjophthalmol-2015-307206 26598576 PMC4975846

[52] O’Colmain, U, et al. 2020. Long-term visual and treatment outcomes of whole-population pre-school visual screening (PSVS) in children: A longitudinal, retrospective, population-based cohort study. Eye, 34(12): 2315–2321. DOI: 10.1038/s41433-020-0821-4 32099079 PMC7785023

[53] Office for National Statistics (ONS). 2022a. Health state life expectancies by national deprivation quintiles, Wales: 2018 to 2020, 25 April 2022. Available at https://www.ons.gov.uk/peoplepopulationandcommunity/healthandsocialcare/healthinequalities/bulletins/healthstatelifeexpectanciesbynationaldeprivationdecileswales/2018to2020 [Last accessed 3 August 2022].

[54] Office for National Statistics. 2022b. Health state life expectancies by national deprivation deciles, England: 2018 to 2020, 25 April 2022. Available at https://www.ons.gov.uk/peoplepopulationandcommunity/healthandsocialcare/healthinequalities/bulletins/healthstatelifeexpectanciesbyindexofmultipledeprivationimd/2018to2020 [Last accessed 21 June 2022].

[55] Orton, E, et al. 2013. Equity of uptake of a diabetic retinopathy screening programme in a geographically and socio-economically diverse population. Public Health, 127(9): 814–821. DOI: 10.1016/j.puhe.2013.04.015 23973045

[56] Pascual, M, et al. 2014. Risk factors for amblyopia in the Vision In Preschoolers study. Ophthalmology, 121(3): 622–629.e1. DOI: 10.1016/j.ophtha.2013.08.040 24140117 PMC3943664

[57] Public Health England (PHE). 2018. Chapter 5: Inequalities in health, 11 September 2018. Available at https://www.gov.uk/government/publications/health-profile-for-england-2018/chapter-5-inequalities-in-health [Last accessed 3 August 2022].

[58] Public Health England. 2019. Place-based approaches for reducing health inequalities: Main report. Available at: https://www.gov.uk/government/publications/health-inequalitiesplace-based-approaches-to-reduce-inequalities/place-based-approaches-for-reducing-health-inequalitiesmain-report [Last accessed 17 November 2020].

[59] Public Health England. 2021a. Health profile for England 2021. Available at https://fingertips.phe.org.uk/static-reports/health-profile-for-england/hpfe_report.html [Last accessed 3 August 2022].

[60] Public Health England. 2021b. Characteristics of women who stop smoking in pregnancy, September 2021. Available at https://assets.publishing.service.gov.uk/government/uploads/system/uploads/attachment_data/file/1019945/Analysis_characteristics_of_women_who_stop_smoking_in_pregnancy.pdf [Last accessed 10 January 2024].

[61] Rahi, JS, Cumberland, PM and Peckham, CS. 2009. Visual function in working-age adults: Early life influences and associations with health and social outcomes. Ophthalmology, 116(10): 1866–1871. DOI: 10.1016/j.ophtha.2009.03.007 19560208

[62] Rahi, JS, Cumberland, PM and Peckham, CS. 2011. Myopia over the lifecourse: Prevalence and early life influences in the 1958 British birth cohort. Ophthalmology, 118(5): 797–804. DOI: 10.1016/j.ophtha.2010.09.025 21185080

[63] Rathore, M, et al. 2023. Measures of multiple deprivation and visual field loss in glaucoma clinics in England: Lessons from big data. Eye, 37: 3615–3620. DOI: 10.1038/s41433-023-02567-z 37165010 PMC10686257

[64] Relton, SD, et al. 2022a. Associations with baseline visual acuity in 12,414 eyes starting treatment for neovascular AMD. Eye, 37(8): 1652–1658. DOI: 10.1038/s41433-022-02208-x 36028762 PMC10219991

[65] Relton, S, et al. 2022b. Associations with visual acuity outcomes after 12 months of treatment in 9401 eyes with neovascular AMD. BMJ Open Ophthalmology, 7(1): e001038. DOI: 10.1136/bmjophth-2022-001038 PMC9240889 36161843

[66] Saxby, E, et al. 2022. Is there an association of socioeconomic deprivation with acute primary angle closure? Eye, 36(6): 1246–1252. DOI: 10.1038/s41433-021-01615-w 34117395 PMC8193016

[67] Scanlon, PH, et al. 2008. Diabetic retinopathy and socioeconomic deprivation in Gloucestershire. Journal of Medical Screening, 15(3): 118–121. DOI: 10.1258/jms.2008.008013 18927093

[68] Schneider, P, et al. 2022. Socioeconomic inequalities in HRQoL in England: An age-sex stratified analysis. Health and Quality of Life Outcomes, 20(1): 121. DOI: 10.1186/s12955-022-02024-7 35918765 PMC9347153

[69] Scottish Government (SG). 2020. Scottish index of multiple deprivation 2020. Available at https://www.gov.scot/collections/scottish-index-of-multiple-deprivation-2020/ [Last accessed 10 January 2024].

[70] Shah, S, et al. 2021a. Diabetic retinopathy in newly diagnosed type 2 diabetes mellitus: Prevalence and predictors of progression; A national primary network study. Diabetes Research and Clinical Practice, 175: 108776. DOI: 10.1016/j.diabres.2021.108776 33753173

[71] Shah, V, Stokes, J and Sutton, M. 2021b. Inequalities in health-related quality of life: repeated cross-sectional study of trends in general practice survey data. British Journal of General Practice, 71(704): e178–e184. DOI: 10.3399/BJGP.2020.0616 PMC7909931 33619049

[72] Sharma, HE, et al. 2014. The role of social deprivation in severe neovascular age-related macular degeneration. British Journal of Ophthalmology, 98(12): 1625–1628. DOI: 10.1136/bjophthalmol-2014-304959 24997180

[73] Sherwin, JC, et al. 2012. Uncorrected refractive error in older British adults: The EPIC-Norfolk Eye Study. British Journal of Ophthalmology, 96(7): 991–996. DOI: 10.1136/bjophthalmol-2011-301430 22535330 PMC4624257

[74] Shweikh, Y, et al. 2015. Measures of socioeconomic status and self-reported glaucoma in the UK Biobank cohort. Eye, 29(10): 1360–1367. DOI: 10.1038/eye.2015.157 26315700 PMC4815692

[75] Sim, PY, et al. 2018. Investigation of factors associated with the success of adult strabismus surgery from the patient’s perspective. Journal of American Association for Pediatric Ophthalmology and Strabismus, 22(4): 266–271.e3. DOI: 10.1016/j.jaapos.2018.03.006 30003957

[76] Simons, K and Preslan, M. 1999. Natural history of amblyopia untreated owing to lack of compliance. British Journal of Ophthalmology, 83(5): 582–587. DOI: 10.1136/bjo.83.5.582 10216059 PMC1723047

[77] Smith, LK, et al. 1995. Factors affecting treatment compliance in amblyopia. Journal of Pediatric Ophthalmology & Strabismus, 32(2): 98–99. DOI: 10.3928/0191-3913-19950301-09 7629678

[78] Social Value Portal (SVP). 2017. Indices of multiple deprivation in the UK. Available at https://socialvalueportal.com/resources/guide/measurement-implementation/indices-of-multiple-deprivation-in-the-uk/ [Last accessed 10 January 2024].

[79] Sukumar, S, et al. 2009. The influence of socioeconomic and clinical factors upon the presenting visual field status of patients with glaucoma. Eye, 23(5): 1038–1044. DOI: 10.1038/eye.2008.245 18688262

[80] UK National Screening Committee (UKNSC). 2023. Child vision screening pathway. Available at https://www.gov.uk/government/publications/child-vision-screening-providing-screening/child-vision-screening-pathway [Last accessed 10 January 2024].

[81] University of Liverpool. n.d. Library Home Page. Available at https://libguides.liverpool.ac.uk/library/ [Last accessed 3 January 2024].

[82] Vassilev, ZP, et al. 2015. Diabetes, cardiovascular morbidity, and risk of age-related macular degeneration in a primary care population. Investigative Ophthalmology & Visual Science, 56(3): 1585–1592. DOI: 10.1167/iovs.14-16271 25670489

[83] Waqar, S, et al. 2012. Cost implications, deprivation and geodemographic segmentation analysis of non-attenders (DNA) in an established diabetic retinopathy screening programme. Diabetes & Metabolic Syndrome: Clinical Research & Reviews, 6(4): 199–202. DOI: 10.1016/j.dsx.2012.08.009 23199538

[84] Welsh Government (WG). 2023. Welsh Index of Multiple Deprivation: index guidance. Available at https://www.gov.wales/welsh-index-multiple-deprivation-index-guidance [Last accessed 10 January 2024].

[85] Williams, C, et al. 2008. Prevalence and risk factors for common vision problems in children: Data from the ALSPAC study. British Journal of Ophthalmology, 92(7): 959–964. DOI: 10.1136/bjo.2007.134700 18480306

[86] Wilson, R and Winnard, Y. 2022. Causes, impacts and possible mitigation of non-attendance of appointments within the National Health Service: A literature review. Journal of Health Organization and Management, 36(7): 892–911. DOI: 10.1108/JHOM-11-2021-0425 35918282

[87] Wong, TL, et al. 2023. The relationship between multiple deprivation and severity of glaucoma at diagnosis. Eye, 37(16): 3376–3381. DOI: 10.1038/s41433-023-02508-w 36959313 PMC10035976

[88] Yip, JLY, et al. 2013. Area deprivation, individual socioeconomic status and low vision in the EPIC-Norfolk Eye Study. Journal of Epidemiology and Community Health, 68(3): 204–210. DOI: 10.1136/jech-2013-203265 24179053 PMC4157999

[89] Yip, JLY, et al. 2015. Area deprivation and age related macular degeneration in the EPIC-Norfolk Eye Study. Public Health, 129(2): 103–109. DOI: 10.1016/j.puhe.2014.10.012 25687711 PMC4357435

[90] Yip, JLY, et al. 2021. Socioeconomic risk factors and age-related macular degeneration in the UK Biobank study. BMJ Open Ophthalmology, 6(1): e000585. DOI: 10.1136/bmjophth-2020-000585 PMC7907888 33693059

